# Dietary fiber alters immunity and intestinal barrier function of different breeds of growing pigs

**DOI:** 10.3389/fimmu.2023.1104837

**Published:** 2023-02-14

**Authors:** Sujuan Ding, Yating Cheng, Md. Abul Kalam Azad, Haibo Dong, Jun He, Pan Huang, Xiangfeng Kong

**Affiliations:** ^1^ Key Laboratory of Agro-ecological Processes in Subtropical Region, Hunan Provincial Key Laboratory of Animal Nutritional Physiology and Metabolic Process, Institute of Subtropical Agriculture, Chinese Academy of Sciences, Changsha, Hunan, China; ^2^ College of Advanced Agricultural Sciences, University of Chinese Academy of Sciences, Beijing, China; ^3^ Institute of Animal Nutrition, Sichuan Agricultural University, Chengdu, Sichuan, China

**Keywords:** dietary fiber, Chinese indigenous pig, Duroc pig, immune function, intestinal barrier function

## Abstract

**Introduction:**

Dietary fiber (DF) regulates immune response and barrier function by interacting with epithelial cells and immune cells. However, the differences in the regulation of intestinal health of different pig breeds by DF remain obscure.

**Methods:**

A total of 60 healthy pigs (20 pigs/breed) from Taoyuan black (TB), Xiangcun black (XB), and Duroc (DR) pigs (body weight = 11.00 ± 1.00 kg) were fed two different levels (low and high) of DF for 28 days to evaluate the differences in the modulation of intestinal immunity and barrier function by DF in different pig breeds.

**Results:**

TB and XB pigs had higher plasma Eos level, Eos%, and Lym% but lower Neu level compared with the DR pigs when fed low DF (LDF). The TB and XB pigs had higher plasma Eos, MCV, and MCH levels and Eos% while lower Neu% compared with the DR pigs when fed high DF (HDF). HDF decreased IgA, IgG, IgM, and sIgA concentrations in the ileum of TB and XB pigs compared with the DR pigs, while the plasma IgG and IgM concentrations of TB pigs were higher than those of the DR pigs. Moreover, compared with the DR pigs, HDF decreased the levels of IL-1β, IL-17, and TGF-β in the plasma, and IL-1β, IL-2, IL-6, IL-10, IL-17, IFN-γ, TGF-β, and TNF-α in the ileum of TB and XB pigs. However, HDF did not affect the mRNA expression of cytokines in the ileum of TB, XB, and DR pigs, while HDF increased the TRAF6 expression of TB pigs compared with the DR pigs. In addition, HDF increased the *Claudin* abundance of TB and DR pigs compared with the pigs feeding with LDF. Moreover, in the LDF and HDF groups, the XB pigs had higher protein abundances of Claudin and ZO-1 compared with the TB and DR pigs.

**Conclusions:**

DF regulated the TB and DR pigs’ plasma immune cells, the XB pigs showed enhanced barrier function, and the DR pigs had increased ileal inflammation, which indicates that Chinese indigenous pigs are more DF tolerant than the DR pigs.

## Introduction

The investigation and commercial interest in swine nutrition have been enormously focused on the gastrointestinal function and optimal regulation of immune function, especially during the post-weaning period, which is the intensive period of using antibiotics to prevent and treat intestinal diseases (1). The gastrointestinal tract is the main site for digestion and absorption of nutrients from diets and is also the largest endocrine and immune organ of the body (2). The host’s innate immune system is the first line of defense against pathogenic invasion. The intestine includes physical barriers and immune cytokines secreted by the intestinal mucosa. Innate immune cells can produce natural antibodies and activate classical pathways. Moreover, innate immune cells constitute important mechanisms of innate immunity and initiate adaptive immune responses mediated by T and B cells (3). Meanwhile, adaptive immune responses have emerged and evolved to provide better protection to the organism against invading pathogens (4). The intestinal physical barrier consists of the mucosal layer, epithelial cells, and non-epithelial mucosal cells (5). Tight junctions between epithelial cells are an important component of the intestinal barrier. The immunoglobulins and cytokines involve in the mucosal immune response and play important roles in maintaining intestinal health (6). Therefore, understanding the changes in the intestinal barrier and immunity in pigs has important implications for regulating intestinal health.

Dietary fiber (DF) negatively affects nutrient digestion, absorption, and energy deposition in the intestine of monogastric animals; thus, it has been considered an anti-nutritional factor for a long time (7). However, substantial evidence suggests that DF can directly provide energy to the host and indirectly participates in gut health and immune function (8, 9). The microbial metabolites produced by the fermentation of DF in the distal small intestine and large intestine interact with various environmental factors of the intestine to affect the composition of intestinal microbes, tissue morphology, and immune function (10). For example, butyrate, produced by the fermentation of intestinal microbes, can enhance intestinal barrier function and maintain physical barrier integrity by stimulating goblet cell differentiation and mucus production (11). Moreover, short-chain fatty acids can promote the differentiation of intestinal regulatory T cells, which maintain intestinal homeostasis by suppressing effector T cells to increase interleukin (IL)-10 production and play an important role in preventing excessive inflammation (12, 13). Therefore, with the continuous discovery of the nutritional value of DF, its functional roles in improving the intestinal health of animals have attracted more attention.

Taoyuan black (TB) and Xiangcun black (XB) pigs are the typical representatives of Chinese indigenous pigs with roughage tolerance. However, how DF regulates the intestinal health of TB and XB pigs is still unknown. Moreover, the differences in the regulation of intestinal health of different pig breeds by DF remained obscure. Therefore, TB, XB, and Duroc (DR) pigs during the growing period were selected to evaluate the effects of DF on the immunity and intestinal barrier function of pigs.

## Materials and methods

### Animals and experimental protocol

A total of 60 healthy TB, XB, and DR pigs (20 pigs from each breed) during the growing stage with an average body weight of 11.00 ± 1.00 kg were randomly selected for this trial. After seven days of adaptation, 30 pigs (10 pigs from each breed) were fed with the Chinese indigenous pigs’ diet (HDF; high DF diet, 6.86% crude fiber content), and 30 pigs (10 pigs from each breed) were fed with the National Research Council (NRC, 2012) recommended diet (LDF; low DF diet, 3.14% crude fiber content). The composition and nutritional levels of diets for different pig breeds met the Chinese nutrient requirements for swine in China (NY/T65-2004) and NRC-recommended requirements (NRC-2012) (**Table 1**). Each pig was housed in a single pen (1.20 × 0.60 × 1.00 m, length × width × height). The experimental animals were fed thrice daily (at 8:00, 14:00, and 20:00), and water was available *ad libitum* at all times. The experimental room was controlled in a constant temperature range (22−25°C) with smooth ventilation. The experiment lasted 28 days.

**Table 1:** Ingredients and nutrient composition of diets for different pig breeds, % as-fed basis.

Items	Low fiber diet	High fiber diet
Ingredients		
Corn	35.70	25.21
Expanded corn	27.41	20.20
Expanded soybean	8.49	12.30
Soybean meal	18.00	16.40
Fish meal	4.50	5.40
Soybean oil	1.68	3.00
Saccharose	2.00	2.00
Limestone powder	0.50	0.30
Corn starch	0.00	8.70
Wheat bran fiber	0.00	4.90
Calcium hydrophosphate	0.58	0.45
Sodium chloride	0.20	0.20
L-lysine hydrochloride (78%)	0.32	0.30
DL-methionine	0.09	0.08
L-threonine (98.5%)	0.02	0.05
L-tryptophan (98%)	0.01	0.01
Choline chloride	0.15	0.15
Vitamin premix^1^	0.05	0.05
Mineral element premix^2^	0.30	0.30
Total	100.00	100.00
Nutrient levels^3^		
Digestible energy (MJ/kg)	3.49	3.50
Crude protein	19.15	19.19
Crude fiber	3.14	7.38
Calcium	0.73	0.69
Phosphorus	0.39	0.36
Lysine	1.22	1.26
Methionine	0.39	0.40
Methionine + Cystine	0.66	0.66
Threonine	0.70	0.74
Tryptophan	0.21	0.33

^1^Providing the following amounts of vitamins per kilogram of a complete diet on an as-fed basis: vitamin A, 6,000.00 IU; vitamin D_3_, 3,000.00 IU; vitamin E, 24.00 mg; vitamin K_3_, 3.00 mg; vitamin B_1_, 1.50 mg; vitamin B_2_, 6.00 mg; vitamin B_6_, 3.00 mg; vitamin B_12_, 0.02 mg; niacin, 14.00 mg; pantothenic acid, 15.00 mg; folic acid, 1.20 mg; and biotin, 0.15 mg.

^2^Providing the following amounts of minerals per kilogram of a complete diet on an as-fed basis: Fe (Fe_2_(SO_4_)_3_·H_2_O), 100.00 mg; Cu (CuSO_4_), 5.00 mg; Zn (ZnSO_4_·H_2_O), 80.00 mg; Mn (MnSO_4_·H_2_O), 3.00 mg; I (Ca(IO_3_)_2_), 0.14 mg; and Se (Na_2_SeO_3_), 0.25 mg.

^3^Data are the results of chemical analysis conducted in triplicate.

All pigs fasted overnight before sampling. Blood samples were collected from the anterior vena cava into potassium ethylenediamine tetraacetate (K_2_-EDTA) anticoagulant tubes (2/10 mL) for blood hematological parameters analyses and heparin sodium anticoagulant tubes (6/10 mL) to collect plasma by centrifuging at 4°C and 3,500 × *g* for 15 min for analysis of immunoglobulins and cytokines. The pigs were euthanized for sampling after intramuscular injection of Zoletil^@^ 50 (Beijing Lab Anim Tech Develop Co., Ltd., Beijing, China). The samples were collected as follows: the liver and spleen were isolated and weighed; middle portions of ileal tissues (4 cm) were collected, quickly frozen into liquid nitrogen, and stored at −80°C to evaluate immune function-related indexes and gene expressions of related signal molecules.

### Analysis of blood hematological parameters

Blood hematological parameters, including the classification and counting of white blood cells and platelets, and the counting and morphology of red blood cells, were measured by BC-5000VET automatic blood cell analyzer (Shenzhen Mindray Biomedical Electronics Co., Ltd., Shenzhen, China) within 2 h after blood collection.

### Analysis of plasma and ileal immunoglobulin and cytokine contents

The contents of immunoglobulins and cytokines in plasma and ileal tissues were determined by enzyme-linked immunosorbent assay (ELISA) kits (Shanghaikx, Shanghai, China), following the manufacturer’s instructions. The absorbance (OD value) at 450 nm was measured using a microplate reader (Infinite M200 PRO, TECAN, Männedorf, Switzerland). The content of total protein in ileum tissues was detected with the BCA protein assay kit (Beyotime, Shanghai, China), and the final contents of immunoglobulins and cytokines of ileal tissues are expressed in unit protein content in the supernatant after homogenization.

### Analysis of ileal gene expression levels related to immune function

Total RNA from ileum tissues was extracted using the TRIzol reagent (Accurate Biology, Hunan, China). The RNA absorption values at 260 nm and 280 nm were measured using a NanoDrop 2000c (Thermo Fisher Scientific, Waltham, MA, USA), and the A_260_/A_280_ ratio range of 1.8 to 2.1 was considered the appropriate quality of the RNA. Meanwhile, RNA degradation was determined by agarose gel electrophoresis. The reverse transcriptional program was performed at 37°C for 15 min and 95°C for 5 s according to the manufacturer’s protocol (Accurate Biology). The primers used in this study are presented in **Table 2**. β-actin was used as the housekeeping gene for gene normalization. The real-time PCR was performed with SYBR^®^ Green Premix *Pro Taq* HS qPCR Kit (Accurate Biology). The following conditions were conducted for PCR cycling program: initial denaturation at 94°C for 30 s; then denaturation at 94°C for 5 s and annealing at 55°C for 30 s with 40 cycles; and a final extension at 55°C for 30 s, which performed on the LightCycler^®^ 480 II Real-Time PCR System (Roche, Basel, Switzerland). Relative expression levels between different groups were calculated using the 2^-ΔΔCt^ method.

**Table 2:** Primers sequences used in this study.

Gene names	Primer sequence (5′-3′)	Size (bp)
*CD14*	F: CGTTTGTGGAGCCTGGAAG R: TGCGGATGCGTGAAGTTG	125
*Claudin*	F: AAGGACAAAACCGTGTGGGA R: CTCTCCCCACATTCGAGATGATT	93
*IL-1β*	F: AAGAGGGACATGGAGAAGCGATTTG R: TTGTTCTGCTTGAGAGGTGCTGATG	114
*IL-2*	F: AAGCTCTGGAGGGAGTGCTA R: CAACAGCAGTTACTGTCTCATCA	115
*IL-6*	F: TGCCACCTCAGACAAAATGC R: AGGTTCAGGTTGTTTTCTGCC	120
*IL-10*	F: GTCCGACTCAACGAAGAAGG R: GCCAGGAAGATCAGGCAATA	106
*IL-17*	F: GCACACGGGCTGCATCAACG R: TGCAACCAACAGTGACCCGCA	124
*IFN-γ*	F: CCATTCAAAGGAGCATGGAT R: GAGTTCACTGATGGCTTTGC	146
*IRAK1*	F: CAAGGCAGGTCAGGTTTCGT R: TTCGTGGGGCGTGTAGTGT	126
*LBP*	F: GAACACAGCCGAATGGTCTAC R: GGAAGGAGTTGGTGGTCAGT	126
*MD2*	F: TGCAATTCCTCTGATGCAAG R: CCACCATATTCTCGGCAAAT	162
*Mucin 2*	F: CTGTGTGGGGCCTGACAA R: AGTGCTTGCAGTCGAACTCA	98
*MyD88*	F: GATGGTAGCGGTTGTCTCTGAT R: GATGCTGGGGAACTCTTTCTTC	146
*NF-κB*	F: AGTACCCTGAGGCTATAACTCGC R: TCCGCAATGGAGGAGAAGTC	109
*NOD1*	F: CTGTCGTCAACACCGATCCA R: CCAGTTGGTGACGCAGCTT	134
*NOD2*	F: GAGCGCATCCTCTTAACTTTCG R: ACGCTCGTGATCCGTGAAC	105
*Occludin*	F: CAGCAGCAGTGGTAACTTGG R: CAGCAGCAGTGGTAACTTGG	100
*RIP2*	F: CAGTGTCCAGTAAATCGCAGTTG R: CAGGCTTCCGTCATCTGGTT	159
*TLR2*	F: GCAATAATGACACCTTCGCTGAGATTC R: AGATGGCTGATGTTCTGAATTGACCTC	139
*TLR4*	F: AGGACGAAGACTGGGTGAGGAATG R: CCTGGATGATGTTAGCAGCGATGG	126
*TNF-α*	F: ATTCAGGGATGTGTGGCCTG R: CCAGATGTCCCAGGTTGCAT	141
*TRAF6*	F: CAAGAGAATACCCAGTCGCACA R: ATCCGAGACAAAGGGGAAGAA	114
*ZO-1*	F: GCCATCCACTCCTGCCTAT R: CGGGACCTGCTCATAACTTC	148
*β-actin*	F: GATCTGGCACCACACCTTCTACAAC R: TCATCTTCTCACGGTTGGCTTTGG	107

CD14, cluster of differentiation 14; IL, interleukin; IFN-γ, interferon-γ; IRAK1, interleukin-1 receptor-associated kinase 1; LBP, lipopolysaccharide binding protein; MD-2, myeloid differential protein-2; MyD88, myeloid differentiation factor 88; NF-κB, nuclear factor-kappa-B; NOD, nucleotide binding oligomerization domain; RIP2, receptor-interact protein 2; TLR, toll-like receptor; TNF, tumor necrosis factor; TRAF6, tumor necrosis factor receptor-associated factor 6; ZO-1, zonula occludens.

### Protein extraction and western blot analysis

Ileal tissue protein was extracted by RIPA protein lysate. The lysed samples were centrifuged at 10,000 × *g* for 10 min at 4°C, and the protein concentration was determined using the BCA kit (Beyotime Biotechnology, Shanghai, China). Equal amounts of protein samples (5 μg/μL) were added to the loading buffer and boiled for 10 min before loading onto 10%/15% gels (Servicebio, Wuhan, China). Proteins from the gels were electronically transferred to PVDF membranes and incubated through a blocking buffer (Beyotime Biotechnology, Shanghai, China) for 15 min at room temperature.

Membranes were incubated with primary antibody at 4°C overnight (10−16 h). The primary antibodies used are as follows: mouse anti-actin antibody, 1: 5000 (66009-1-Ig, Proteintech, Chicago, IL, USA); rabbit anti-Occludin antibody, 1: 2000 (27260-1-AP, Proteintech); rabbit anti-ZO-1, 1: 2000 (21773-1-AP, Proteintech); rabbit anti-Claudin1, 1: 2000 (13050-1-AP, Proteintech); rabbit anti-Mucin 2, 1: 2000 (ab272692, Abcam, Cambridge, UK).

After incubation of the primary antibody, membranes were washed three times with phosphate buffered saline with Twen-20 (PBST) for 15 min each time, and then incubated in HRP-conjugated goat anti-mouse IgG antibody (AWS0001 and AWS0002, 1: 5000, Abiowell, Changsha, China) diluted in PBST at room temperature for 90 min, and washed three times with PBST for 15 min each time. Finally, membranes were exposed to electrochemiluminescence, and the images were saved for further data analysis.

### Data analysis

All data are expressed as means ± standard error of the mean (SEM). Statistical analysis was performed by a two-way ANOVA for pig breed and DF using the SPSS 22.0 (IBM Corporation, Chicago, USA) software and Tukey’s *post-hoc* multiple comparison test. Individual pigs were considered as the experimental unit. A *P* value < 0.05 was regarded as a significant difference.

## Results

### Effects of dietary fiber on blood hematological parameters in different breeds of pigs

The effects of DF on blood hematological parameters in different breeds of pigs are listed in **Table 3**. In the LDF groups, the neutrophils (Neu) level was lower (*P* < 0.05) in the TB and XB pigs than in the DR pigs, and the neutrophils ratio (Neu%) of XB pigs was lower (*P* < 0.05) than the TB pigs. The eosinophils count (Eos) and eosinophil ratio (Eos%) were higher (*P* < 0.05) in the TB and XB pigs than in the DR pigs; lymphocyte ratio (Lym%) was higher (*P* < 0.05) in the TB and XB pigs than in the DR pigs, whereas absolute value of lymphocytes (Lym) was higher (*P* < 0.05) in the XB pigs than in the DR pigs. In addition, the mean corpuscular volume (MCV) level was higher (*P* < 0.05) in the TB pigs than in the DR pigs. In the HDF groups, the Neu level was lower (*P* < 0.05) in the XB pigs than in the TB and DR pigs, whereas the Eos and mean red blood cell hemoglobin (MCH) levels and Eos% were lower (*P* < 0.05) in the DR pigs than in the XB and TB pigs. The Lym% in the XB pigs was lower (*P* < 0.05) than in the TB and DR pigs whereas was higher (P < 0.05) than in the TB pigs, and the Neu% was higher (*P* < 0.05) in the DR pigs than in the TB and XB pigs. Moreover, the basophil count (Bas) level was lower (*P* < 0.05) in the TB pigs than in the XB and DR pigs, while the MCV level was higher (*P* < 0.05) in the TB and XB pigs than in the DR pigs. However, different levels of DF did not affect the hematological parameters among the same pig breed (*P* > 0.05).

**Table 3:** Effects of dietary fiber on blood hematological parameters in different pig breeds.

Items	Low dietary fiber			High dietary fiber			SEM	*P*-values		
	TB	XB	DR	TB	XB	DR		Fiber	Breed	Fiber × Breed
WBC, 10^^9^/L	24.46	23.63	27.14	26.09	26.29	28.14	1.98	0.281	0.344	0.914
Neu, 10^^9^/L	8.57^BC^	4.94^D^	13.43^A^	8.78^BC^	6.49^CD^	11.29^AB^	1.24	0.902	<.001	0.330
Lym, 10^^9^/L	14.15^BC^	17.19^AB^	12.44^C^	15.58^ABC^	17.83^A^	15.43^ABC^	1.17	0.083	0.010	0.595
Eos, 10^^9^/L	0.27^A^	0.31^A^	0.03^B^	0.38^A^	0.38^A^	0.10^B^	0.06	0.088	<.001	0.916
Bas, 10^^9^/L	0.05^BC^	0.08^ABC^	0.07^ABC^	0.04^C^	0.09^A^	0.09^AB^	0.01	0.418	0.008	0.363
Mon, 10^^9^/L	1.42	1.12	1.17	1.31	1.21	1.31	0.18	0.784	0.539	0.757
Neu%	33.70^B^	21.12^C^	48.05^A^	33.03^B^	23.30^C^	39.69^B^	2.83	0.327	<.001	0.166
Lym%	59.37^B^	72.75^A^	47.25^C^	60.44^B^	70.56^A^	55.12^BC^	3.10	0.378	<.001	0.262
Mon%	5.70	4.65	4.39	4.98	4.47	4.60	0.54	0.601	0.227	0.690
Eos%	1.04^A^	1.22^A^	0.08^B^	1.42^A^	1.36^A^	0.33^B^	0.20	0.115	<.001	0.823
Bas%	0.19	0.26	0.23	0.13	0.29	0.26	0.05	0.972	0.048	0.559
RBC, 10^^12^/L	7.54	7.59	7.81	7.41	7.60	8.16	0.38	0.808	0.378	0.800
HGB, g/L	114.50	112.80	113.40	111.90	117.00	116.30	5.50	0.740	0.940	0.807
HCT, %	36.31	34.79	35.47	34.99	36.32	36.38	1.70	0.789	0.975	0.679
MCV, fL	48.22^A^	46.17^ABC^	45.49^BC^	47.28^AB^	48.10^A^	44.65^C^	0.84	0.941	0.006	0.161
MCH, pg	15.21^AB^	14.94^ABC^	14.55^BC^	15.13^AB^	15.48^A^	14.28^C^	0.28	0.785	0.011	0.339
MCHC, g/L	315.90	323.30	320.00	320.00	321.80	319.68	2.59	0.721	0.212	0.526
RDW-CV, %	20.39	20.96	21.52	21.46	20.96	22.98	0.59	0.086	0.046	0.448
RDW-SD, fL	36.73	36.39	37.49	37.86	37.82	39.36	1.03	0.084	0.387	0.936
PLT, 10^^9^/L	339.80	282.10	251.50	260.00	361.20	345.25	39.13	0.336	0.802	0.057
MPV, fL	8.69	8.29	8.66	8.52	8.57	8.63	0.21	0.881	0.549	0.539
PDW, %	15.24	15.14	14.88	15.14	15.21	14.90	0.11	0.963	0.016	0.747
PCT, %	0.29	0.23	0.22	0.22	0.31	0.30	0.03	0.323	0.921	0.055

Values are presented as means with their SEM, *n* = 10. Different upper-case letters within the same row indicated significant differences among different days of age (*P < 0.05). WBC, white blood cell count; Neu, neutrophils count; Lym, absolute value of lymphocytes; Mon, monocytes count; Eos, eosinophils count; Bas, basophil count; Neu%, neutrophil ratio; Lym%, lymphocyte ratio; Mon%, monocyte ratio; Eos%, eosinophil ratio; Bas%, basophil ratio; RBC, red blood cell count; HGB, hemoglobin; HCT, hematocrit; MCV, mean corpuscular volume; MCH, mean corpuscular hemoglobin; MCHC, mean corpuscular hemoglobin concentration; RDW-CV, coefficient variation of red blood cell volume distribution width; RDW-SD, standard deviation in red cell distribution width; PLT, platelet; MPV, mean platelet volume; PDW, platelet distribution width; PCT, plateletocrit. TB, Taoyuan black pig; XB, Xiangcun black pig; DR, Duroc pig.

### Effects of dietary fiber on plasma and ileal immunoglobulin contents in different breeds of pigs

**Figure 1:**
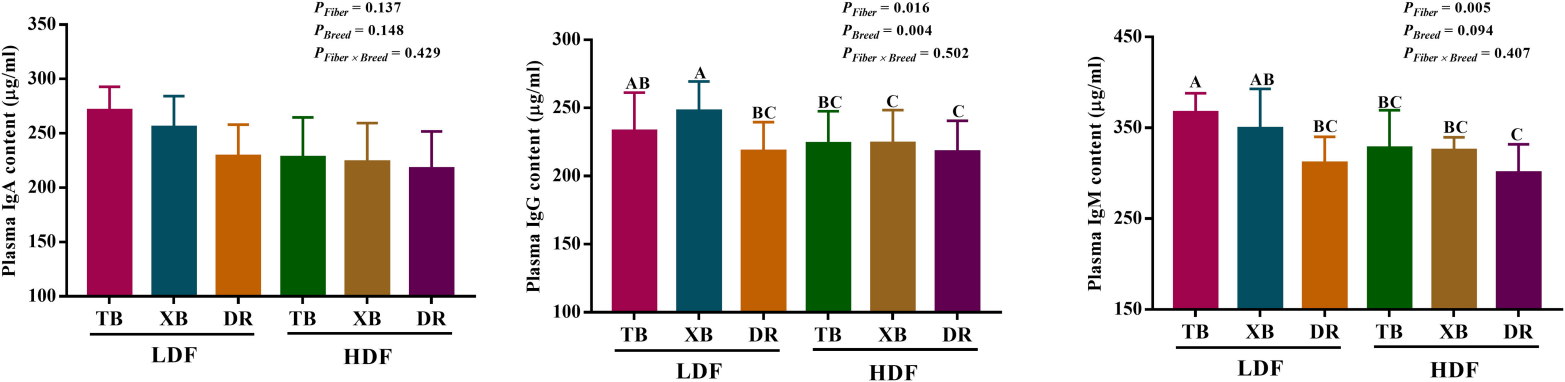
Effects of dietary fiber on plasma immunoglobulin contents in different breeds of pigs. Values are shown as means with their SEM (*n* = 10). Different upper-case letters indicate significant differences among different groups (*P* < 0.05). Ig, immunoglobulin; TB, Taoyuan black pig; XB, Xiangcun black pig; DR, Duroc pig; LDF, low dietary fiber; HDF, high dietary fiber.

The effects of DF on plasma immunoglobulin contents in different breeds of pigs are shown in **Figure 1**. Dietary fiber affected the plasma IgM and IgG contents in three breeds of pigs, but the pig breeds only affected the content of plasma IgG, and there were no interactions between DF and pig breeds. TB pigs fed with LDF had higher (*P* < 0.05) plasma IgM and IgG contents than the DR pigs fed with LDF. The HDF decreased (*P* < 0.05) the plasma IgM and IgG contents of TB pigs and the IgG content of XB pigs compared with the LDF.

**Figure 2:**
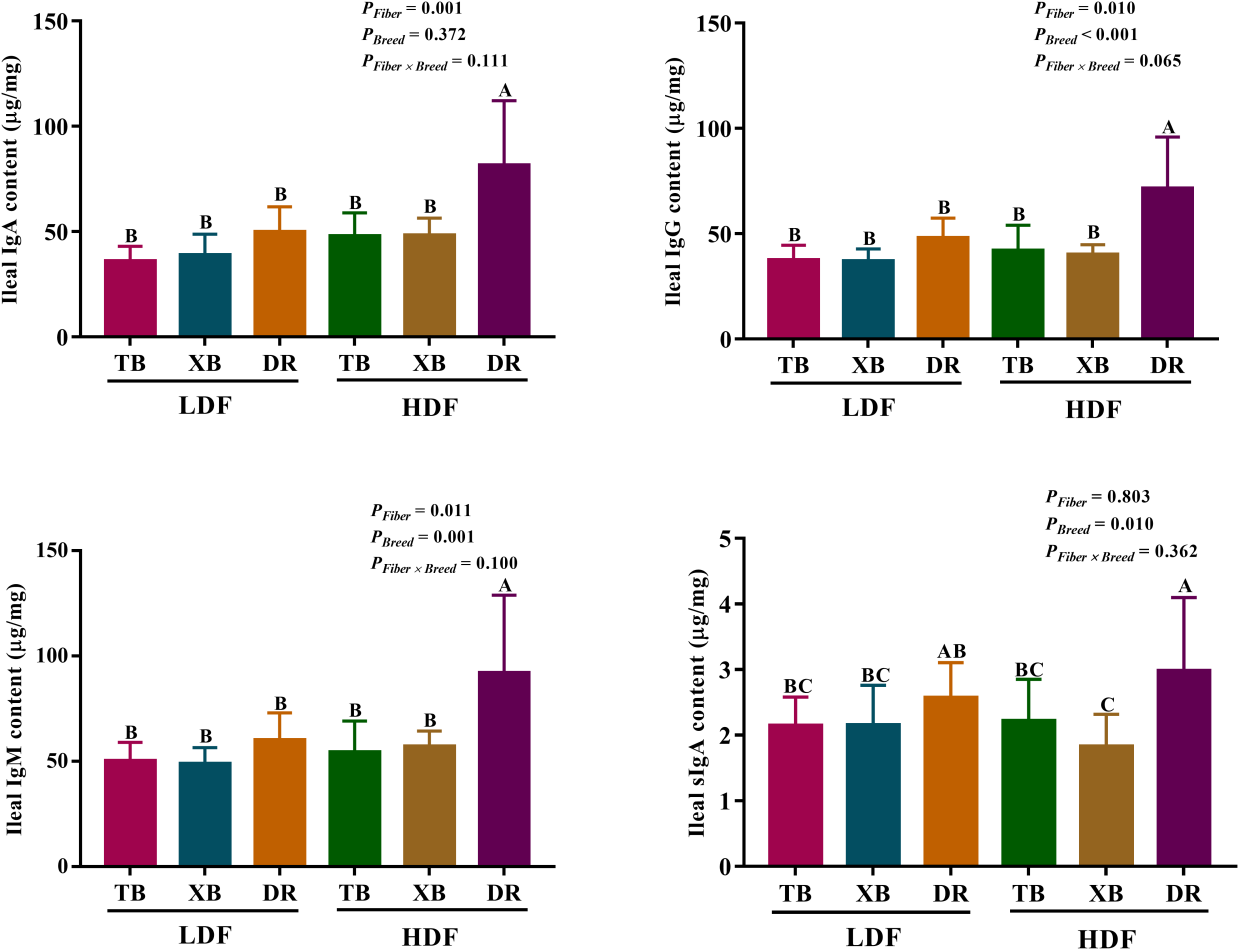
Effects of dietary fiber on ileal immunoglobulin contents in different breeds of pigs. Values are shown as means with their SEM (*n* = 10). Different upper-case letters in the histogram indicate significant differences among different groups (*P* < 0.05). Ig, immunoglobulin; TB, Taoyuan black pig; XB, Xiangcun black pig; DR, Duroc pig; LDF, low dietary fiber; HDF, high dietary fiber.

The effects of DF on ileal immunoglobulin contents in different breeds of pigs are presented in **Figure 2**. Dietary fiber affected the IgA, IgG, and IgM contents, while pig breeds affected the IgG, IgM, and sIgA contents in the ileum. There were no interactions between DF and pig breeds on immunoglobulin contents. The HDF increased (*P* < 0.05) the ileal IgA, IgM, and IgG contents in the DR pigs compared with the LDF. The DR pigs fed with HDF had higher (*P* < 0.05) ileal IgA, IgM, IgG, and sIgA contents than the TB and XB pigs fed with low/high DF.

### Effects of dietary fiber on plasma and ileal cytokines in different breeds of pigs

**Figure 3:**
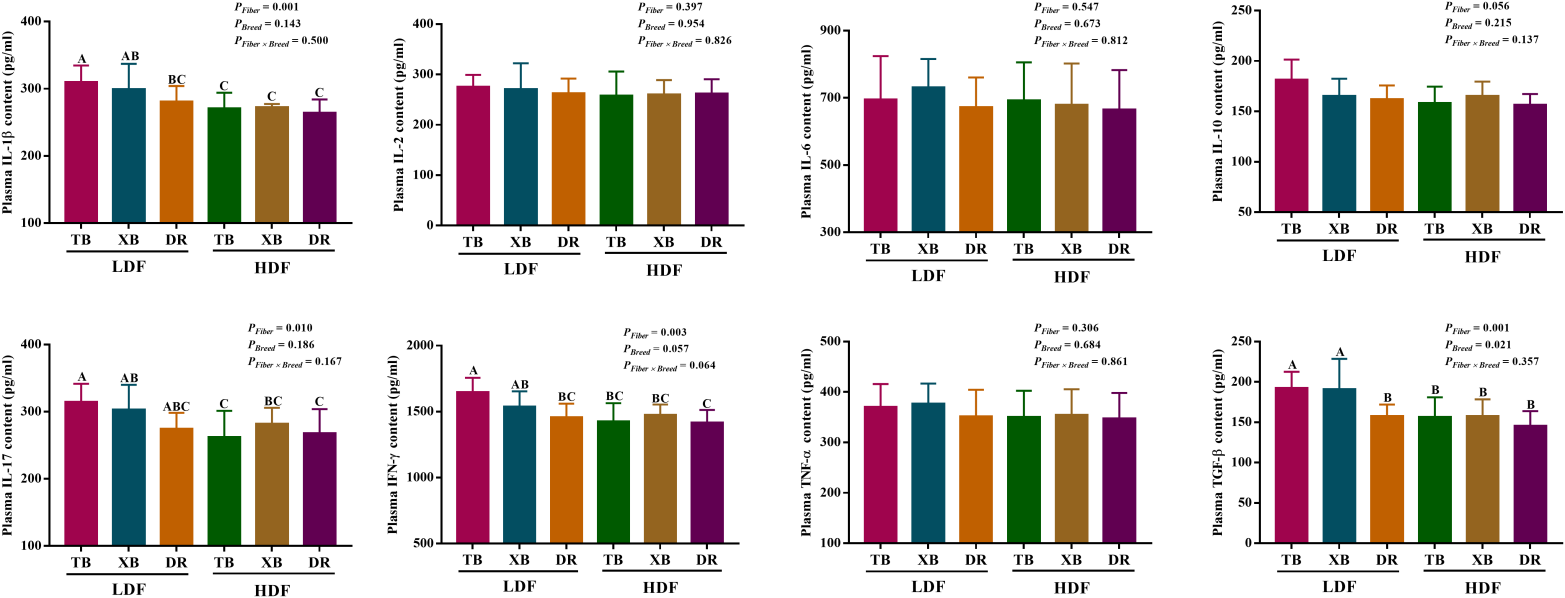
Effects of dietary fiber on plasma cytokine contents in different breeds of pigs. Values are shown as means with their SEM (*n* = 10). Different upper-case letters in the histogram indicate significant differences among different groups (*P* < 0.05).TB, Taoyuan black pig; XB, Xiangcun black pig; DR, Duroc pig; LDF, low dietary fiber; HDF, high dietary fiber; IL, interleukin; IFN, interferon; TNF, tumor necrosis factor, TGF, transforming growth factor.

The effects of DF on plasma cytokines in different breeds of pigs are shown in **Figure 3**. In the LDF groups, the contents of IL-1β and interferon (IFN)- γ were lower (*P* < 0.05) in the DR pigs than in the TB pigs, as well as the transforming growth factor (TGF)-β in the DR pigs than in the TB and XB pigs. The HDF decreased (*P* < 0.05) the contents of IL-1β, IL-17, IFN-γ, and TGF-β in the TB pigs and IL-1β, IL-17, and TGF-β in the XB pigs compared with the pigs fed with LDF, respectively.

**Figure 4:**
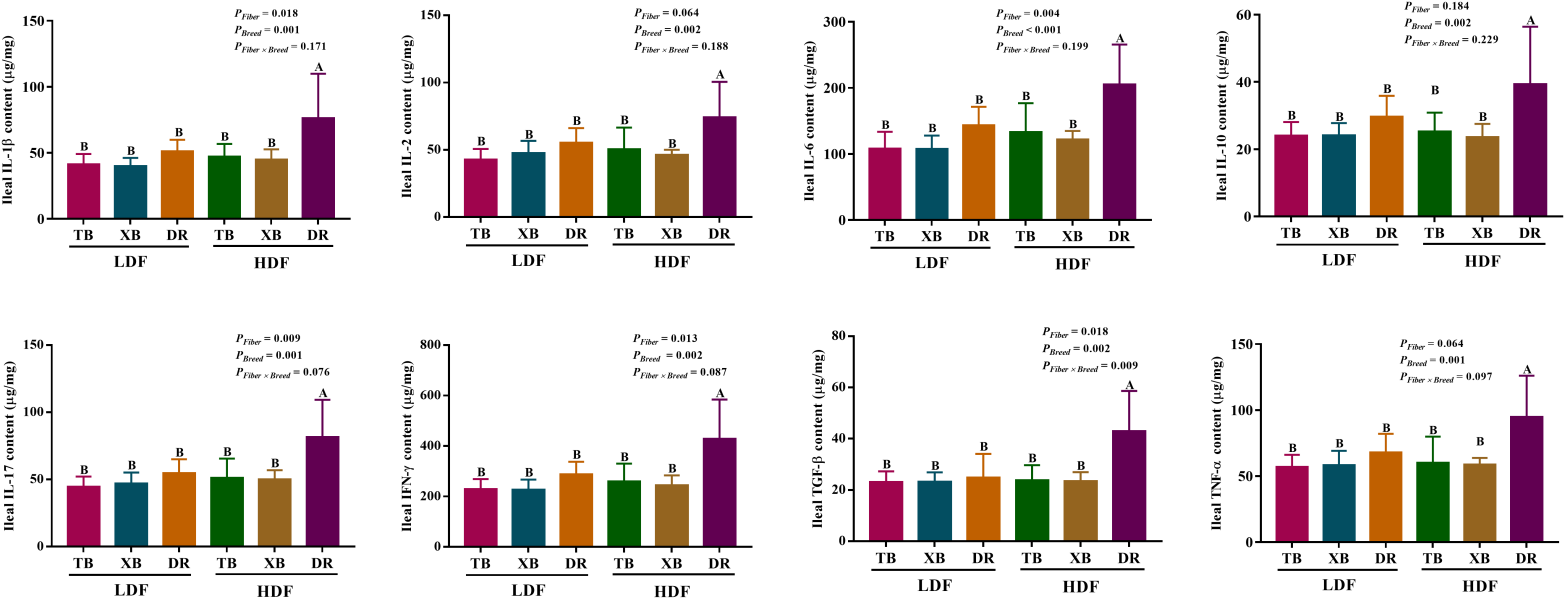
Effects of dietary fiber on ileal cytokine contents in different breeds of pigs. Values are shown as means with their SEM (*n* = 10). Different upper-case letters in the histogram indicate significant differences among different groups (*P* < 0.05). TB, Taoyuan black pig; XB, Xiangcun black pig; DR, Duroc pig; LDF, low dietary fiber; HDF, high dietary fiber; IL, interleukin; IFN, interferon; TNF, tumor necrosis factor, TGF, transforming growth factor.

The effects of DF on ileal cytokines in different breeds of pigs are shown in **Figure 4**. Multivariate analysis of variance showed that DF had an effect on cytokines contents of DR pigs, and there were differences in cytokines contents among the pig breeds (*P* < 0.05), but DF and breed interactions did not affect the cytokine contents of pigs (*P* > 0.05). The HDF increased (*P* < 0.05) the contents of IL-1β, IL-2, IL-6, IL-10, IL-17, IFN- γ, TGF-β, and TNF-α in the DR pigs than in the TB and XB pigs, and the DR pigs fed with LDF.

### Effects of dietary fiber on the expression level of genes related to immune function in different breeds of pigs

**Figure 5:**
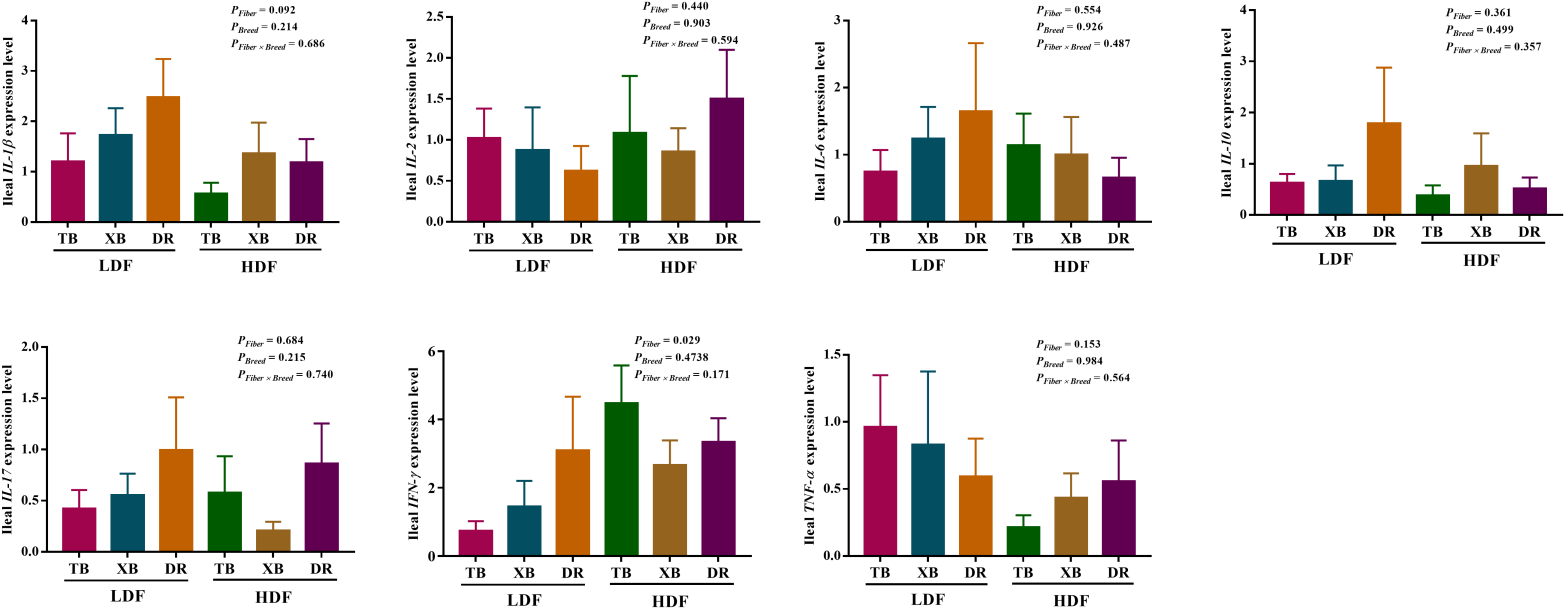
Effects of dietary fiber on expression level of genes related to immune function in different breeds of pigs. Values are shown as means with their SEM (*n* = 10). Different upper-case letters in the histogram indicate significant differences among different groups (*P* < 0.05).TB, Taoyuan black pig; XB, Xiangcun black pig; DR, Duroc pig; LDF, low dietary fiber; HDF, high dietary fiber; IL, interleukin; IFN, interferon; TNF, tumor necrosis factor, TGF, transforming growth factor.

**Figure 6:**
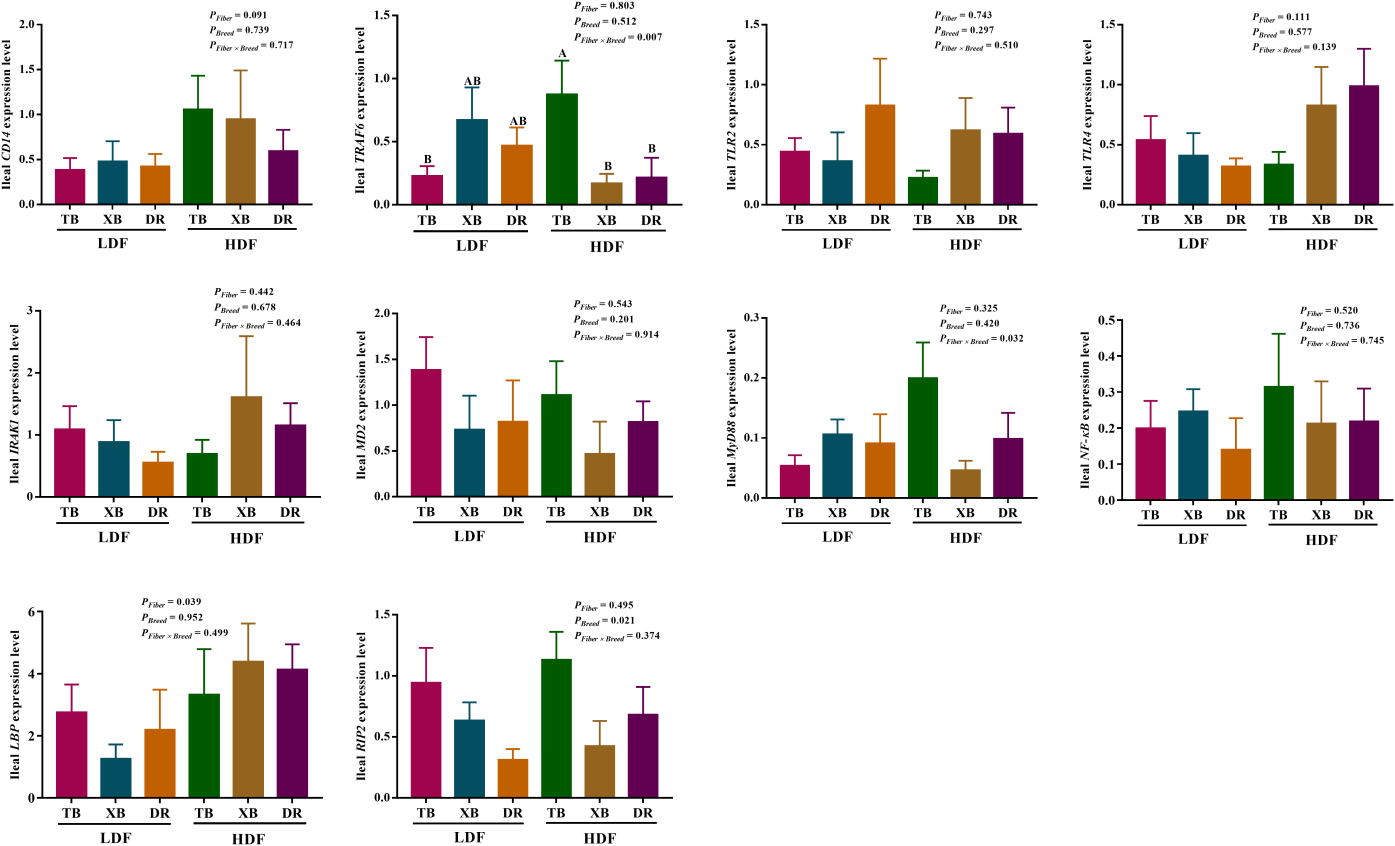
Effects of dietary fiber on expression level of genes involved in immune regulatory pathways in different breeds of pigs. Values are shown as means with their SEM (*n* = 10). Different upper-case letters in the histogram indicate significant differences among different groups (*P* < 0.05).TB, Taoyuan black pig; XB, Xiangcun black pig; DR, Duroc pig; LDF, low dietary fiber; HDF, high dietary fiber; CD14, cluster of differentiation 14; IRAK1, interleukin-1 receptor-associated kinase 1; LBP, lipopolysaccharide binding protein; MD-2, myeloid differential protein-2; MyD88, myeloid differentiation factor 88; NF-κB, nuclear factor-κ-B; NOD, nucleotide binding oligomerization domain; RIP2, receptor-interact protein 2; TLR, toll-like receptor; TNF, tumor necrosis factor; TRAF6, tumor necrosis factor receptor-associated factor 6.

The effects of DF on the expression level of genes related to immune function in different breeds of pigs are shown in **Figures 5**, **6**. The HDF increased (*P* < 0.05) the tumor necrosis factor receptor-associated factor 6 (*TRAF*6) expression of TB pigs compared with the XB and DR pigs fed with LDF. Moreover, HDF increased (*P* < 0.05) the *TRAF6* expression of TB pigs compared with the TB pigs fed with LDF. However, DF levels did not affect the expression level of genes related to the immune function in the same pig breed (*P* > 0.05).

### Effects of dietary fiber on the expression level of genes and abundance of proteins related to ileal barrier function in different breeds of pigs

**Figure 7:**
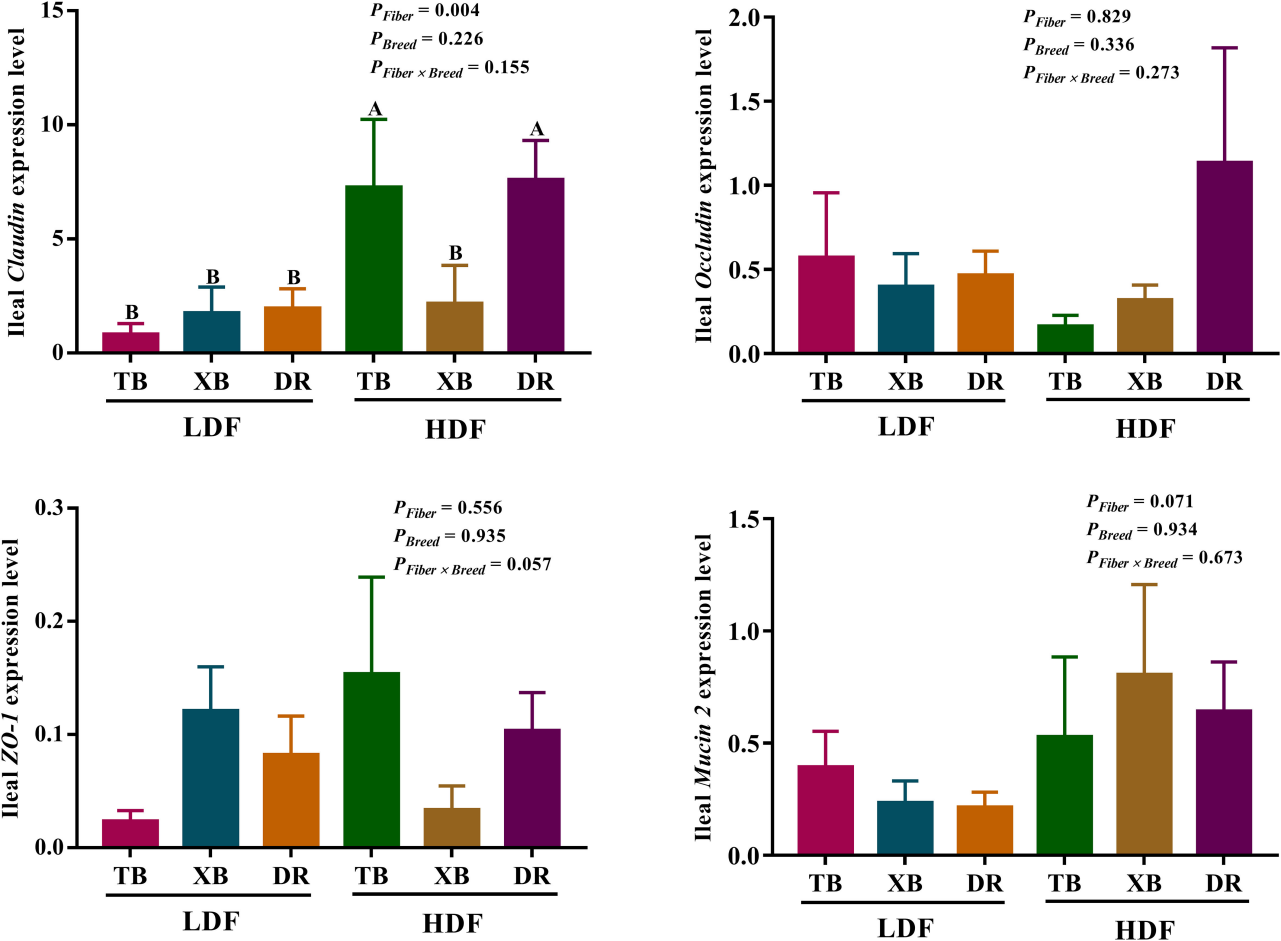
Effects of dietary fiber on expression level of genes related to ileal barrier function in different breeds of pigs. Values are shown as means with their SEM (*n* = 10). Different upper-case letters in the histogram indicate significant differences among different groups (*P* < 0.05). TB, Taoyuan black pig; XB, Xiangcun black pig; DR, Duroc pig; LDF, low dietary fiber; HDF, high dietary fiber; ZO, zonula occludens.

The effects of DF on the expression level of genes related to ileal barrier function in different breeds of pigs are presented in **Figure 7**. The HDF increased (*P* < 0.05) the *Claudin* expression of TB and DR pigs compared with the pigs fed with LDF. In the HDF groups, the *Claudin* expression of XB pigs was lower (*P* < 0.05) than the TB and DR pigs. Different levels of DF did not affect other gene expressions in the same pig breed (*P* > 0.05).

**Figure 8:**
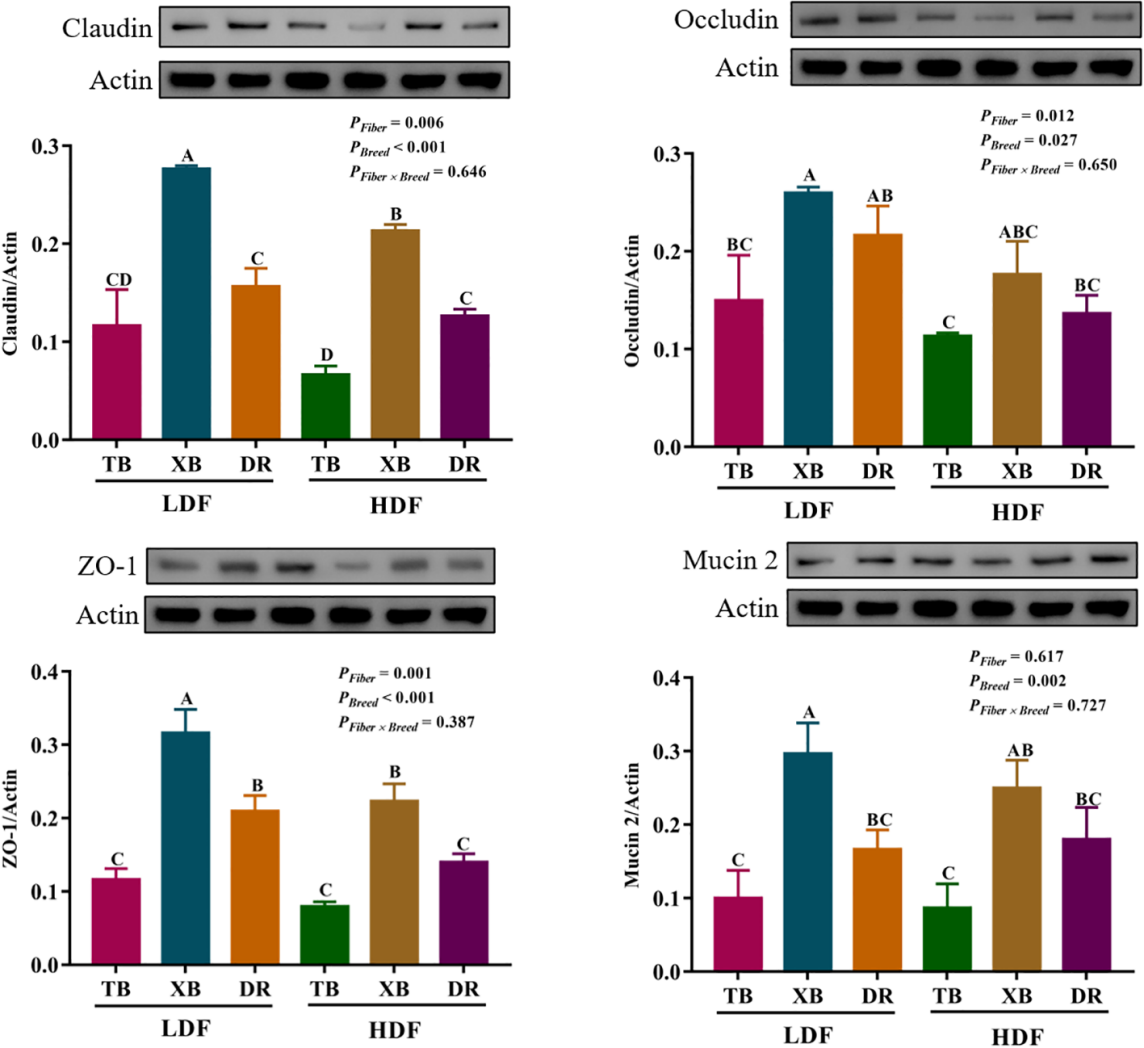
Effects of dietary fiber on abundance of proteins related to ileal barrier function in different breeds of pigs. Values are shown as means with their SEM (*n* = 3). Different upper-case letters in the histogram indicate significant differences among different groups (*P* < 0.05).TB, Taoyuan black pig; XB, Xiangcun black pig; DR, Duroc pig; LDF, low dietary fiber; HDF, high dietary fiber; ZO, zonula occludens.

The effects of DF on the abundance of proteins related to ileal barrier function in different breeds of pigs are shown in **Figure 8**. The HDF decreased (*P* < 0.05) the Claudin abundance of XB pigs and the zonula occludens (ZO)-1 abundance of XB and DR pigs compared with the pigs fed with LDF. In the LDF groups, the XB pigs had higher (*P* < 0.05) Claudin, ZO-1, and Mucin 2 abundances compared with the TB and DR pigs, as well as Occludin abundance compared with the TB pigs. In the HDF groups, the XB pigs had higher (*P* < 0.05) Claudin and ZO-1 abundances compared with the TB and DR pigs.

### Analysis of the correlation between ileal barrier functional gene and immunoglobulins or cytokines

**Figure 9:**
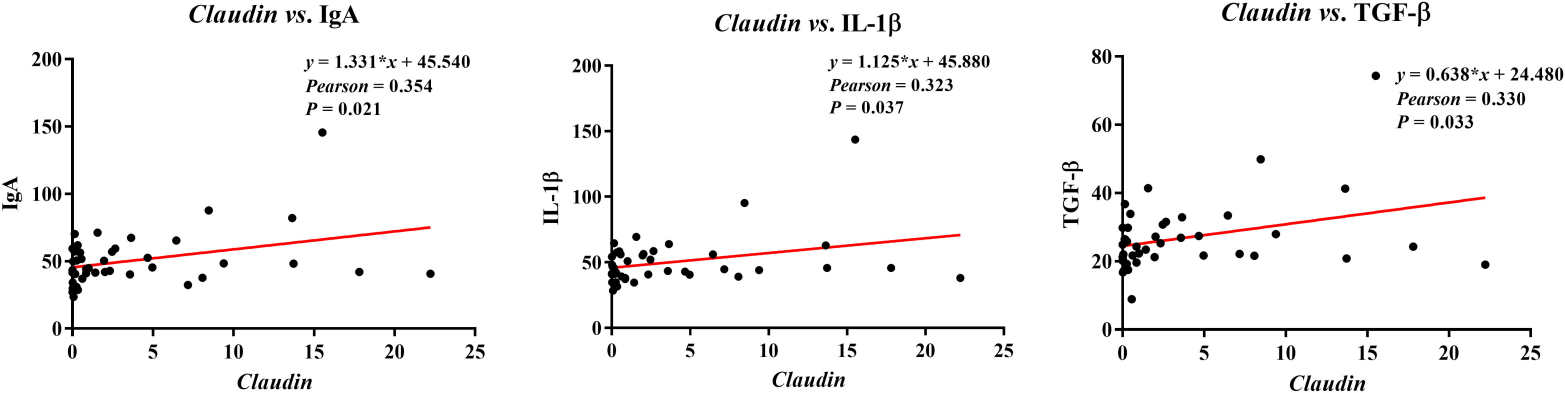
Analysis of the correlation between ileal barrier function gene expressions and immunoglobulins or cytokines. Ig, immunoglobulin; IL, interleukin; TGF, transforming growth factor.

The correlations between ileal barrier function-related gene expressions and immunoglobulins or cytokines contents are presented in **Figure 9**. The *Claudin* expression was negatively correlated with plasma contents of IgA (*Pearson* = 0.354, *P* = 0.021), IL-1β (*Pearson* = 0.323, *P* = 0.037), and TGF-β (*Pearson* = 0.330, *P* = 0.033).

## Discussion

Dietary fiber (DF) consists of various natural compounds (14). In traditional nutrition studies, DF is considered as an anti-nutritional factor because it cannot be degraded by endogenous digestive enzymes and reduces the digestibility of nutrients. Therefore, a low percentage of DF is generally used in monogastric animal diets (15). However, DF can stimulate the growth of intestinal microbiota by maintaining the anaerobic environment and inhibiting the propagation of facultative anaerobic bacteria in the intestine (16, 17). Meanwhile, microbial metabolites produced by the intestinal microbiota through anaerobic fermentation of fiber directly provide energy for intestinal cells or indirectly participate in regulating the immune and barrier functions to maintain intestinal health (9). Currently, the application of DF in pig nutrition is still challenging, as various factors need to be considered, such as the wide range of DF raw material sources, the complexity of its composition, and pig breeds. Therefore, the present study compared the effects of DF on intestinal immunity and barrier function of different breeds of pigs. The findings demonstrated that Chinese indigenous pigs have higher fiber tolerance than the DR pigs, and supplementation of HDF improved the intestinal mechanical barrier function in the TB and DR pigs.

The cells in the immune system circulate between the central and peripheral lymphoid organs and migrate to the damaged parts through the transportation of blood to play their function (18). Neutrophils are polymorphonuclear leukocytes with powerful antibacterial functions and are one of the important regulators of acute inflammation (19). Under inflammation conditions, neutrophils participate in the damage site to mediate inflammatory response (20). Early evidence has reported that neutrophils participated in inflammatory sites in weaned piglets exposed to enterotoxigenic *Escherichia coli* (ETEC), highly correlated with ETEC and prone to intense respiratory bursts (21). The present study revealed that HDF increased Neu% of DR pigs than LDF; moreover, HDF increased Neu% in the TB and XB pigs compared with the DR pigs, suggesting that the DF caused a higher degree of intestinal damage in DR Pigs than the TB and XB pigs, and the TB and XB pigs have the better adaptation to HDF.

The intestinal mucosa releases immunoglobulins to maintain intestinal homeostasis (22). For example, IgA secreted from intestinal lamina propria is translocated across intestinal epithelial cells (IECs) through cellular receptor polymeric immunoglobulin receptor and plays an important role in the mucosal surface adaptive immune response (23). Moreover, sIgA plays a crucial role in regulating intestinal microbiota composition, protecting IECs from pathogen invasion, and supporting immune system development (24). The present study showed that DF had no effects on the plasma immunoglobulin content of different breeds of pigs; however, HDF increased the ileal IgA, IgM, and IgG contents of DR pigs compared with the TB, XB, and DR pigs fed with LDF and TB and XB pigs fed with HDF, indicating that DF may cause more intestinal damage in DR pigs than the TB and XB pigs. Previous studies reported that oral administration of porcine IgG accelerated the removal of hemolytic bacteria from the feces of weaned piglets infected with *Escherichia coli* (25).

Cytokines are proteins secreted by intestinal cells that regulate intestinal homeostasis by regulating intestinal cell death, proliferation, and inflammatory response to pathogens (26). It has been reported that the piglets’ diet enriched with 60.8% fiber increased the plasma IL-10 content but did not affect TNF-α, IL-1β, IL-6, and IL-10 contents, suggesting that DF can improve the anti-inflammatory response of weaned piglets (27). The present study showed that HDF decreased the plasma IL-1β, IL-17, and TGF-β contents of TB and XB pigs, whereas HDF increased the ileal IL-1β, IL-2, IL-6, IL-10, IL-17, IFN-γ, TGF-β, and TNF-α contents in the DR pigs. Previous studies showed that DF did not affect the growth performance of commercial piglets, but increased the *IL-6*, *IL-1β*, and *IL-10* expressions in ileal tissues (28). Research evidence has found that DF may elicit an intestinal immune response in pigs, while the findings of the present study suggest that the TB and XB pigs may be more adaptive to HDF than the DR pigs, and the mechanism may be related to the immune regulation of metabolites produced by the roughage feeding tolerance of Chinese indigenous pigs.

The crosstalk of nuclear factor kappa-B (NF-κB) and mitogen-activated protein kinase (MAPK) plays an important role in the feedback mechanism of immunity (29). TLRs signaling activates NF-κB and MAPKs *via* the adaptor protein MyD88 or toll/interleukin-1 receptor (TIR) domain-containing adaptor inducing IFN-β (TRIF) to regulate the expression of several proinflammatory cytokines, including *IL-1*, *IL-6*, *TNF-α*, and *IFN-γ* (30). A previous study reported that the ileal *TRAF6, NF-κB*, and *MyD88* expressions were up-regulated in piglets exposed to *Clostridium perfringens* infection, suggesting that the *MyD88/NF-κB* signaling pathway is closely related to host immune response induced by infection (31). The present study showed that the TB pigs fed with HDF had lower ileal *TRAF* expression than the XB and DR pigs but did not affect the ileal *MyD88*/*NF-κB* pathway. The TRAF is an important signaling molecule that transduces cytokines on the cell surface, which is involved in IL/TLRs signal transduction and activation of inflammatory and apoptotic signaling pathways (32). These findings suggest that DF may mediate ileal inflammation and apoptosis through the IL/TLRs signaling pathway.

The IECs act as a barrier to close contact with the external environment, and their integrity is to maintain intestinal health (33). The intestinal barrier promotes the invasion of pathogenic bacteria, leading to an immune response and increased susceptibility to infection (34). Intestinal tight junctions (TJs) proteins, including Claudin, ZO-1, and Occludin, allow intercellular water ions and molecular communication between macromolecules, which play a potential role in maintaining the intestinal barrier (35). In the present study, HDF could improve the mRNA expression of ileal *Claudin* in TB and DR pigs, but decrease the protein abundances of ileal Claudin and ZO-1 in XB and DR pigs. The reason for the inconsistency of gene expression and protein abundance may be that the degradation of mRNA caused by the diet led to the reduction of protein translation level. Previous study suggested that nutritional status affects the gene transcription rate by affecting the changes of acetyl-CoA carboxylase and fatty acid synthase, thereby affecting mRNA levels and ultimately enzyme protein synthesis (36). These findings suggest that dietary fiber may induce a decrease in enzymes related to protein synthesis, but the specific regulatory mechanisms need to be further explored. In addition, the protein abundances of ileal Claudin and ZO-1 of XB pigs is higher than those of TB and DR pigs regardless of the DF, suggesting XB pigs showed a hybrid advantage in the intestinal barrier function under DF tolerance. Animal studies showed that DF, as a fermentation substrate of gut microbes, produces short-chain fatty acids. Microbial metabolites not only provide energy for IECs and participate in intestinal immune responses, but also provide a molecular basis for regulating the integrity of the intestinal barrier (37). For example, butyrate can maintain the integrity of the intestinal barrier by regulating mucin expression. Propionic acid alleviates DSS-induced intestinal injury by improving the *ZO-1* and *Occludin* expressions in colon tissues (38). Therefore, intestinal microbes regulate the intestinal barrier function through metabolites such as short-chain fatty acids produced by DF fermentation.

Intestinal barrier function was affected by immunoglobulins or cytokines. Research evidence revealed that M cell mediated by *Claudin 4* specific targeting peptide can enhance mucosal IgA response, which provides new insights for targeting ligand-dependent mucosal vaccination strategies (39). Moreover, cytokine-mediated TJs dysfunction results in increased paracellular permeability, such as IL-1β inhibits Occludin protein levels in Caco-2 cells (40). A previous study demonstrated that TGF-β increased the permeability of epithelial cells, and exposure of rat hepatocytes to TGF-β resulted in decreased Claudin 1 protein abundance (41). The present study showed that the ileal *Claudin* expression positively correlated with ileal IgA, IL-1β, and TGF-β contents, suggesting that the ileal TJs function of pigs was affected by immunoglobulins and cytokines; however, the specific mechanism needs to be further explored.

## Conclusion

In summary, HDF regulated the composition of immune cells, increased immunoglobulins in the ileum of DR pigs and ileal TJs of XB pigs, and decreased the plasma TGF-β content of TB and XB pigs. However, the contents of proinflammatory and anti-inflammatory cytokines were increased in the ileum of DR pigs. These findings suggest that there were differences in immune responses between Chinese indigenous and foreign pig breeds, which might be associated with the excellent characteristics of roughage feeding tolerance of the Chinese indigenous pigs, and the utilization of DF of indigenous pig breeds was better than that of the foreign pig breed.

## Data availability statement

The original contributions presented in the study are included in the article/Supplementary Materials. Further inquiries can be directed to the corresponding author.

## Ethics statement

The procedures of animal experiments were carried out in accordance with the Animal Care and Use Committee of the Institute of Subtropical Agriculture, Chinese Academy of Sciences (Approval no. 20200018).

## Author contributions

Writing – Original draft preparation, SD. Animal feeding – SD, and YC. Index detection – SD and PH. Writing – Review and editing, MA, HD, and XK. Trial Protocol – XK and JH. Research Fund Support – XK and SD. All authors contributed to the manuscript revision, read, and approved the submitted version.
